# Supplementary material for: Design of buried charged networks in artificial proteins
Source: Nat Commun. 2021 Mar 25;12:1895. doi: 10.1038/s41467-021-21909-7 (PMC7994573; doi:10.1038/s41467-021-21909-7)
Supplement: Supplementary file 1 — Supplementary Information [file 41467_2021_21909_MOESM1_ESM.pdf]

Supplementary Information

Design of Buried Charged Networks in Artificial Proteins

Baumgart *et al.*

Content

Supplementary Figure 1 | Structural stability of *Maquette* 1.
Supplementary Figure 2 | Spectroscopic and biophysical characterisation of *Maquette* 1 and 2.
Supplementary Figure 3 | Structural stability of *Maquette* 2.
Supplementary Figure 4 | Statistical analysis of natural ion-pairs.
Supplementary Figure 5 | Amino acid occurrence, ion-pair composition, and abundance in the OPM dataset.
Supplementary Figure 6 | Structural characterisation of *Maquette* 3.
Supplementary Figure 7 | Simulation statistics of *Maquette* constructs 1 - 3.
Supplementary Figure 8 | NMR relaxation data of *Maquette* 2.

Supplementary Table 1 | Sequences of *de novo* protein *Maquettes*.
Supplementary Table 2 | NMR and refinement statistics of *Maquette* 2.
Supplementary Table 3 | Crystallographic data collection and refinement statistics on *Maquette* 3.
Supplementary Table 4 | Summary of MD simulations.
Supplementary Table 5 | Summary of primers used.
Supplementary Table 6 | DNA sequences of the *de novo*-protein *Maquettes*.
Supplementary Table 7 | Buffers used for protein purification of *de novo*-protein *Maquettes*.

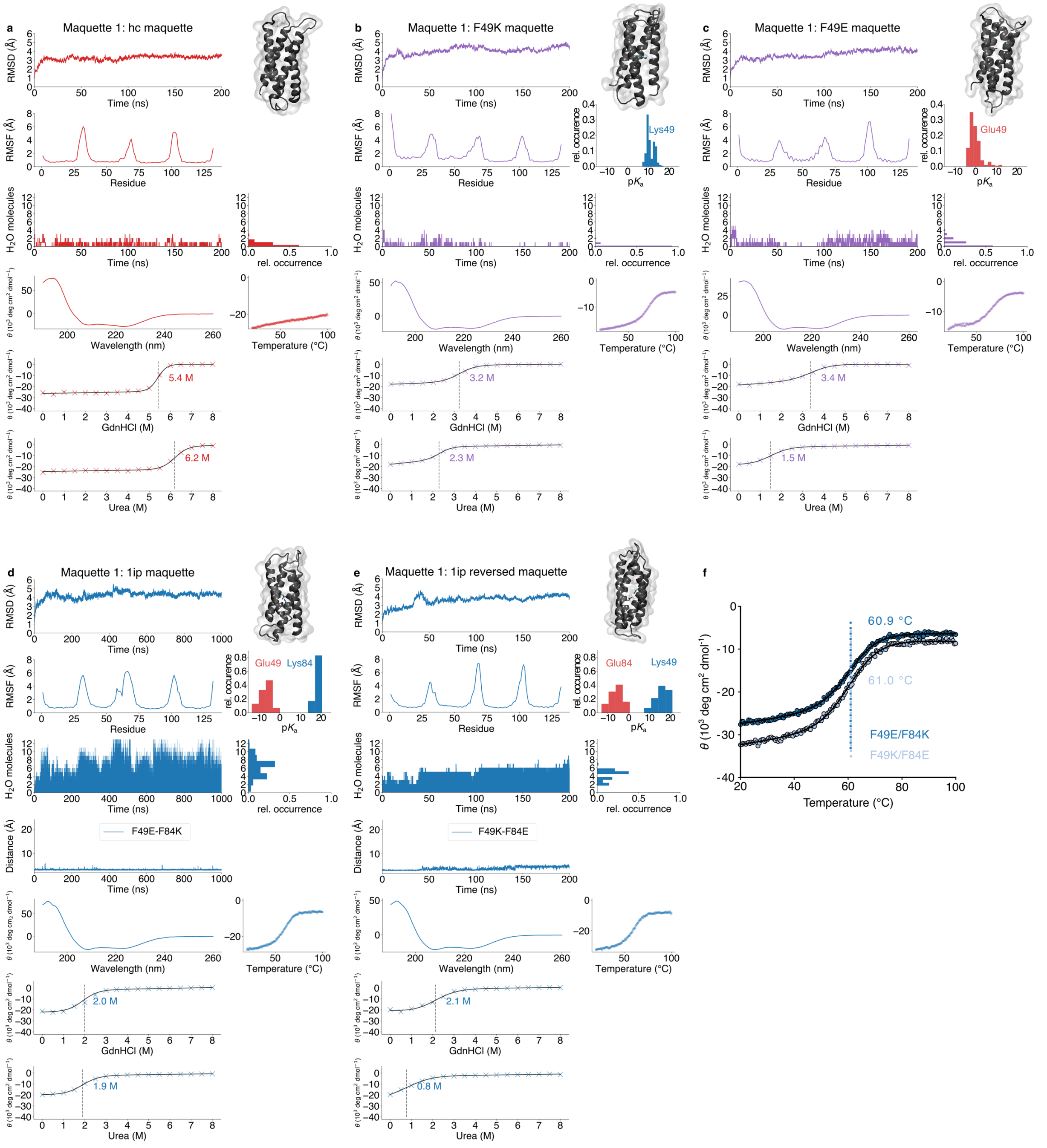

Supplementary Figure 1 | Structural stability of *Maquette* 1. a, *Maquette* 1 with hydrophobic core, **b,** *Maquette* 1 with F49K, **c,** *Maquette* 1 with F49E, **d,** *Maquette* 1 with buried ion-pair (F49E/F84K)**, e,** *Maquette* 1 with reversed buried ion-pair (F49K/F84E). The figure shows (from *top* to *bottom) root-mean-square deviations* (RMSD), *root-mean-square-fluctuations* (RMSF), count of buried water molecules, and predicted pK_a values of charged residues from MD simulations; CD spectra, temperature melting curves, and chemical unfolding profiles with GdnHCl and urea, **f**, temperature melting curves of ip (F49E/F84K, dark blue) and inverted ip (F49K/F84E, light blue). The melting points were obtained from sigmoidal fits, shown as black lines.

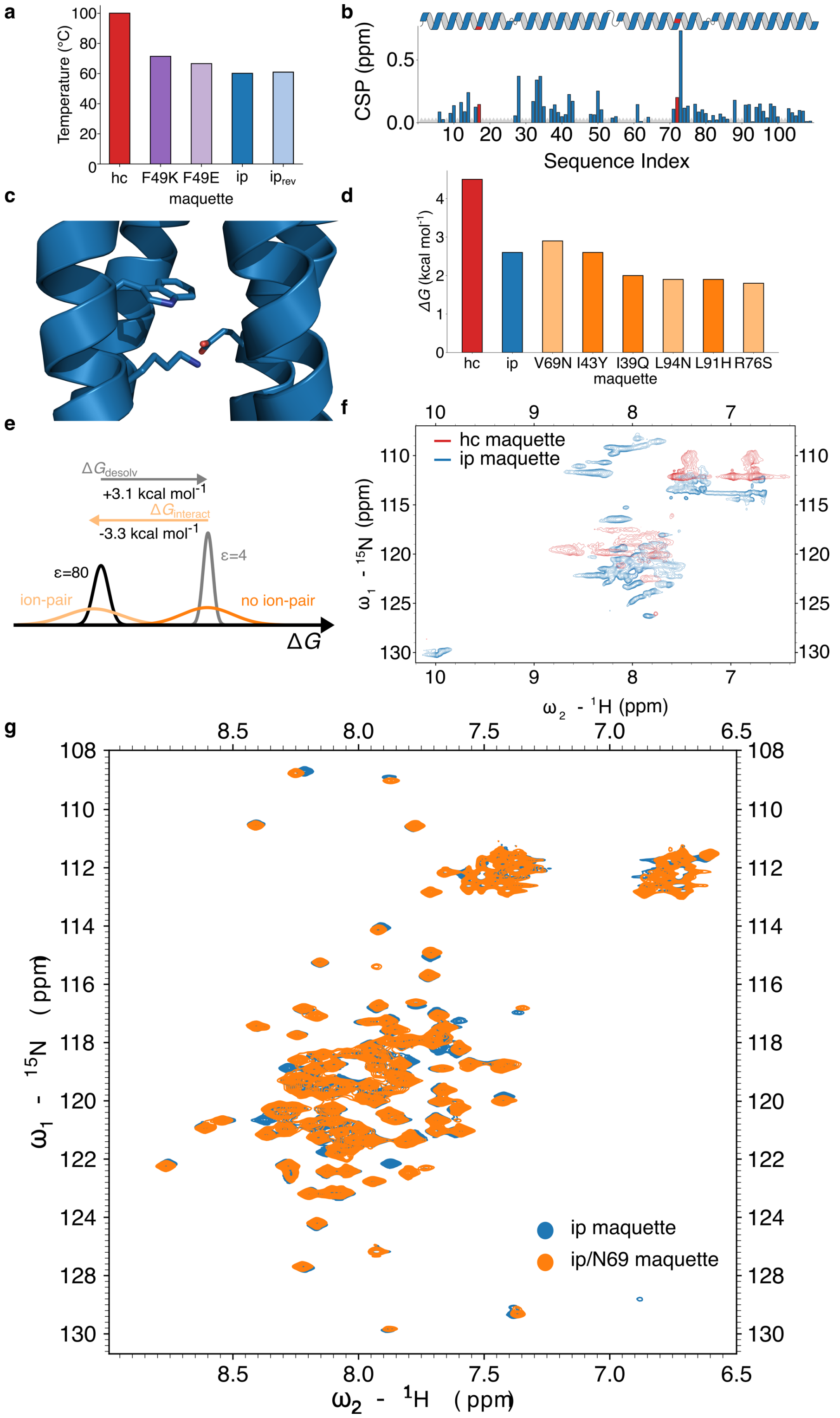

Supplementary Figure 2 | Spectroscopic and biophysical characterisation of *Maquette* 1 and 2. a, Thermal unfolding series of the *Maquette* 1 constructs. **b**, NMR spectroscopic data on *Maquette* 2. Chemical shift perturbation plot between hydrophobic core and ion-pair model. Charged residues are marked in red. **c**, Trp68 stabilises the buried ion-pair in structural models of *Maquette* 2. **d**, Δ*G* derived from chemical unfolding experiments of *Maquette* 2 constructs with a buried ion-pair. **e**, electrostatic model calculations on embedding charge-shielding Asn element into the non-polar protein core without (+3.1 kcal mol^{-1}) and with (-0.2 kcal mol^{-1}) a surrounding ion-pair in *Maquette* 2. **f**, HSQC of hc (red) and ip (blue) of *Maquette* 1. **g**, HSQC of ip/V69N and ip *Maquette* 2.

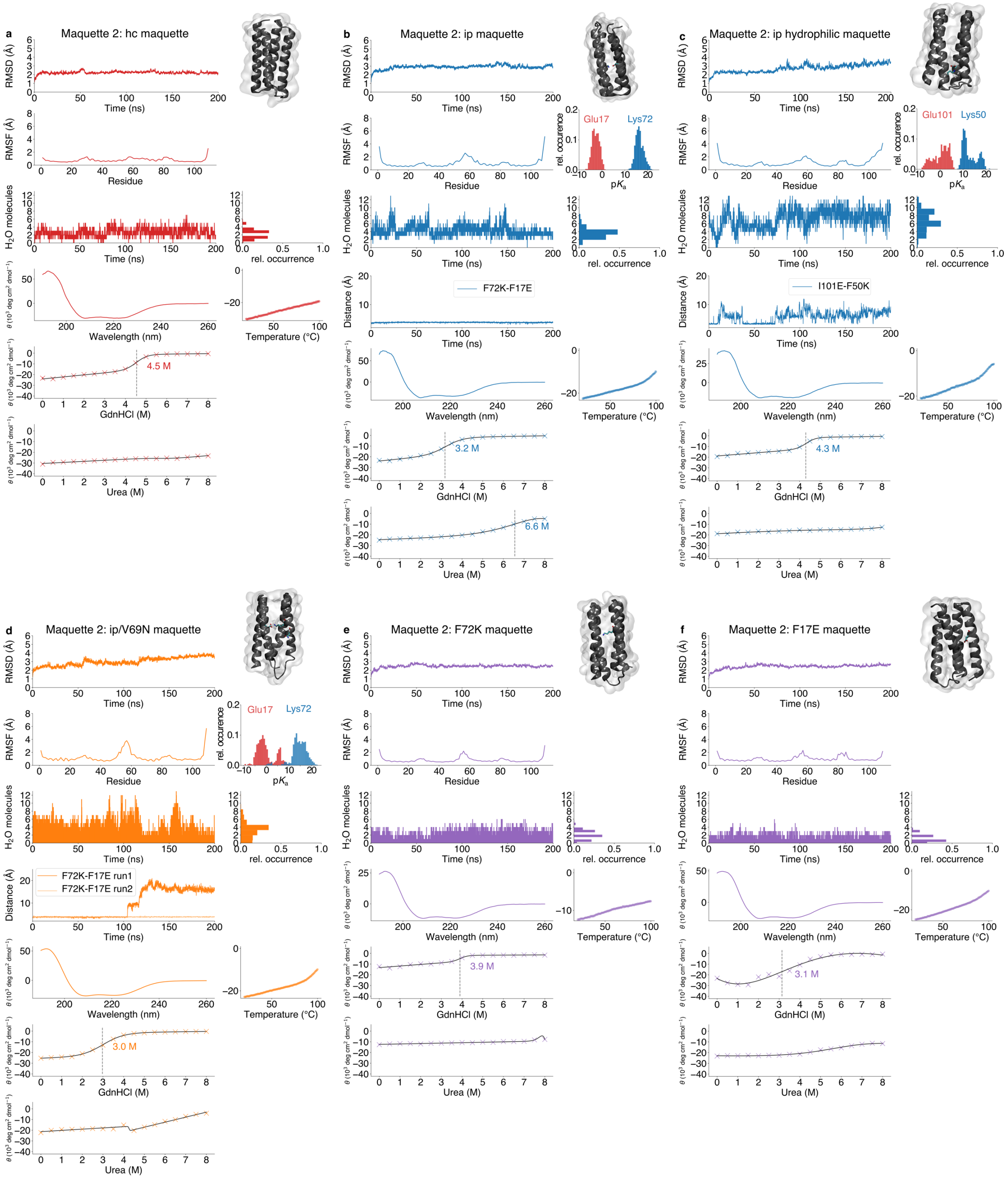

Supplementary Figure 3 | Structural stability of *Maquette* 2. a, *Maquette* 2 with hydrophobic core, **b,** *Maquette* 2 with buried ion-pair (F17E/F72K)**, c**, *Maquette* 2 with ion-pair (I101E/F50K) in hydrophilic part, **d**, *Maquette* 2 ion-pair (F17E/F72K) and stabilising element Asn69. The figure shows (from *top* to *bottom) root-mean-square deviations* (RMSD), *root-mean-square-fluctuations* (RMSF), count of buried water molecules, and predicted pK_a values of charged residues from MD simulations; CD spectra, temperature melting curves, and chemical unfolding profiles with GdnHCl and urea.

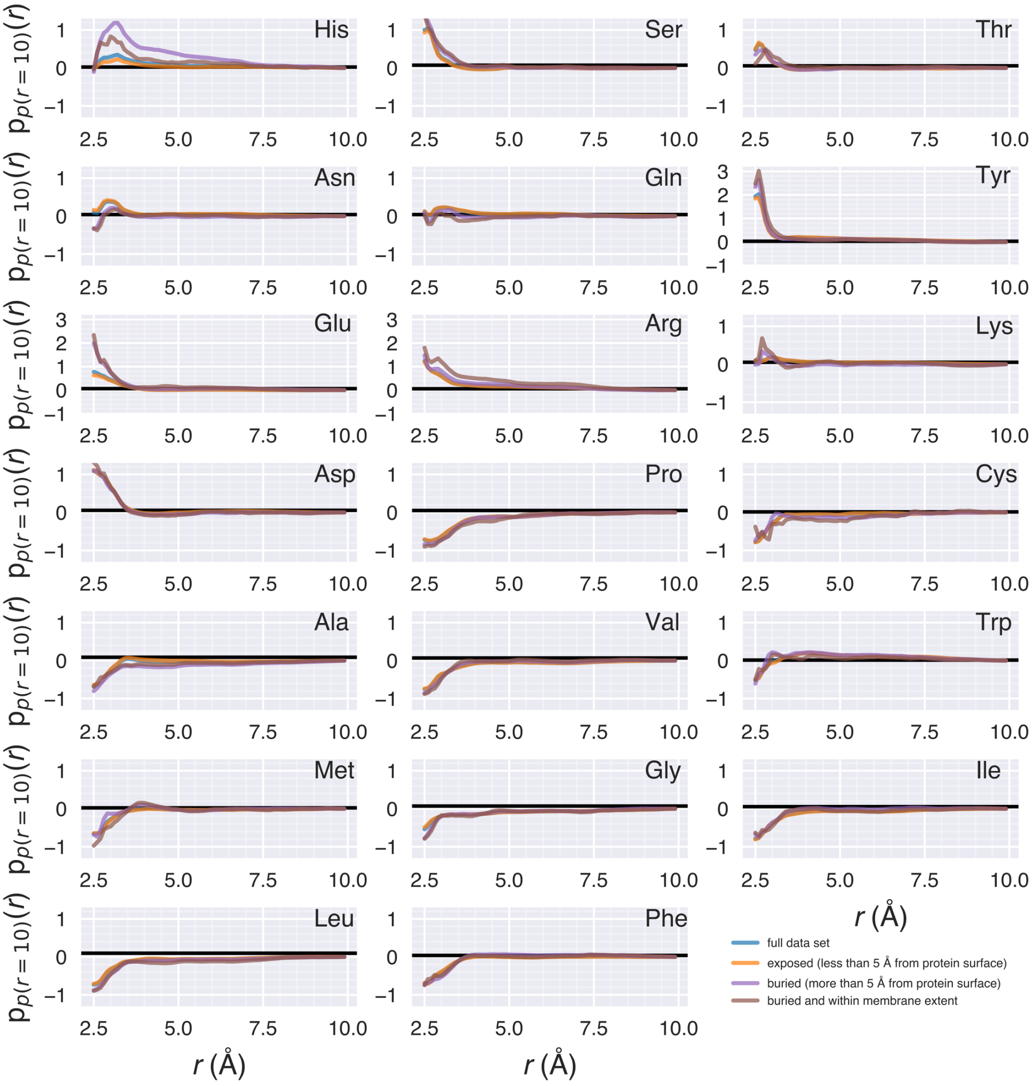

Supplementary Figure 4 | Statistical analysis of natural ion-pairs. Natural occurrence of amino acids around 182,880 ion-pairs in 6,045 membrane proteins as a function of distance relative to the ion-pair.

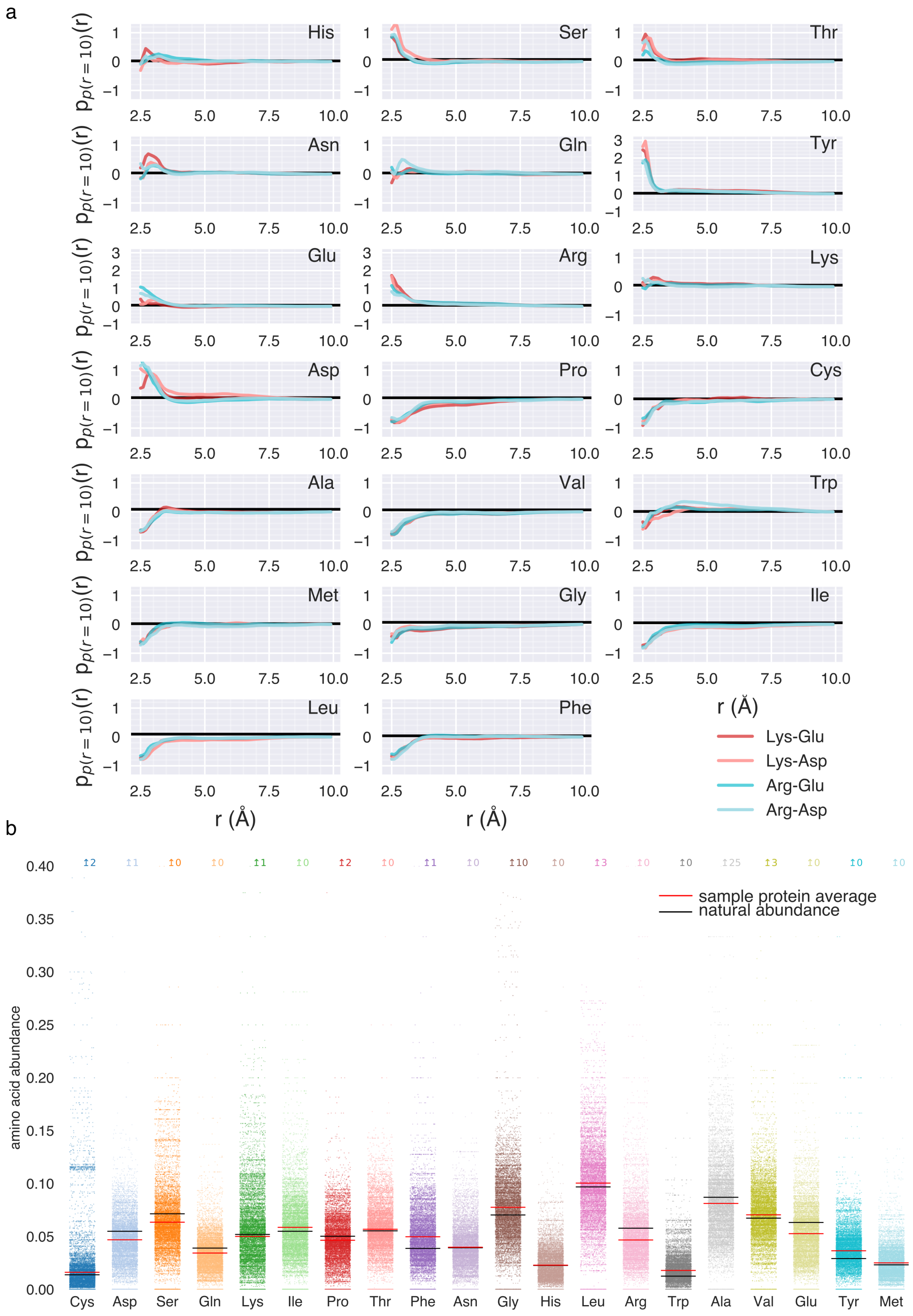

Supplementary Figure 5 | Amino acid occurrence depending on the ion-pair composition and natural abundance of amino acids in the OPM data set. a, Natural occurrence of amino acids around different types of ion-pairs as a function of distance relative to the ion-pair **b,** Natural abundance of amino acids as compared to their abundance within the OPM dataset. Amino acid abundances of proteins within the OPM dataset are shown as dots with the average marked as red lines, whereas individual natural amino acid abundances[1] are shown as black lines. For each residue, the outliers, ↥*N*, with values above 0.4 are not shown.

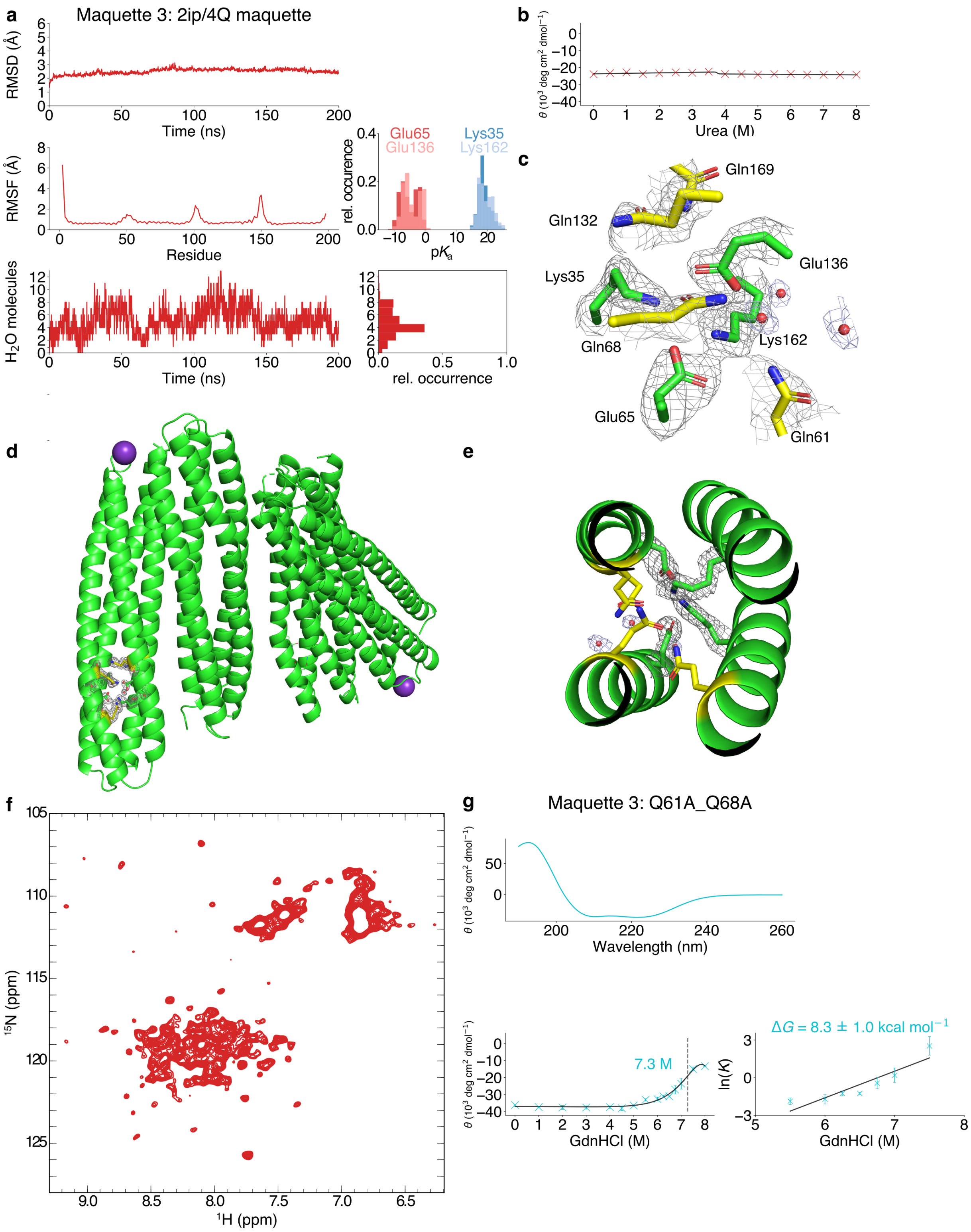

Supplementary Figure 6 | Structural characterisation of *Maquette* 3. a, RMSD, RMSF, predicted pK_a of buried residues, and count of buried water molecules from MD simulations. **b**, chemical unfolding with urea, **c**, structure and electron density maps of the ion-pair region in stick representation, and resolved water molecules that form contacts with Gln61. **d**, The crystallographic packing of *Maquette* 3. A resolved potassium ion is shown as purple *van-der-Waals* spheres. **e**, top view of the buried ion pair region. **f**, HSQC of 2ip/4Q *Maquette* 3. **g**, *Maquette* 3 with 2ip and Q61A/Q68A, CD spectrum, chemical unfolding profile with GdnHCl up to 8M, and ΔG value calculated from chemical unfolding experiments. Data points are presented as mean values of triplicates (n=3 independent experiments) with ± standard deviation as error bars.

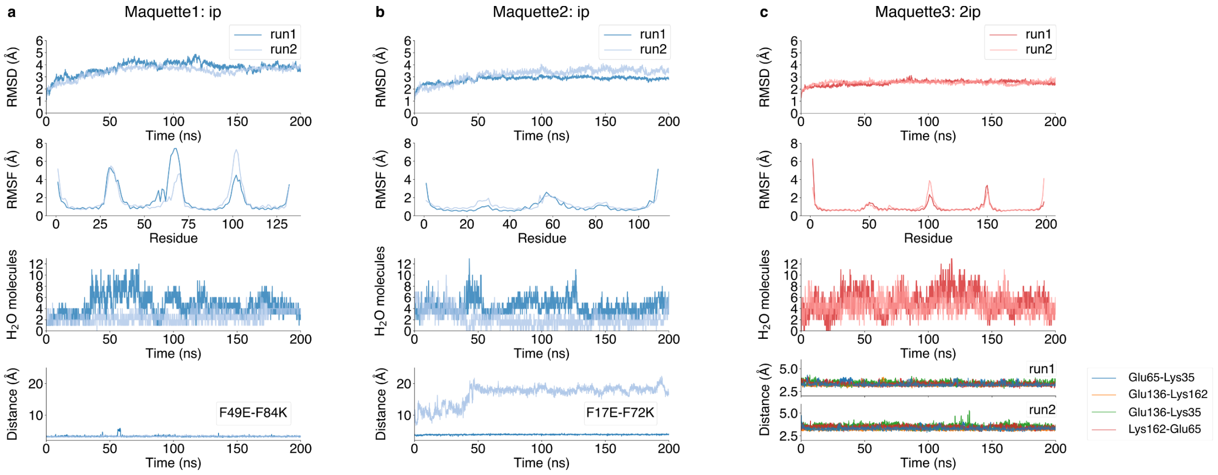

Supplementary Figure 7 | Simulation statistics of *Maquette* constructs 1 - 3. The figure shows RMSD, RMSF, count of buried water molecules and distance of ion-pairs / hydrophobic residues from MD simulations listed in Supplementary Table 4.

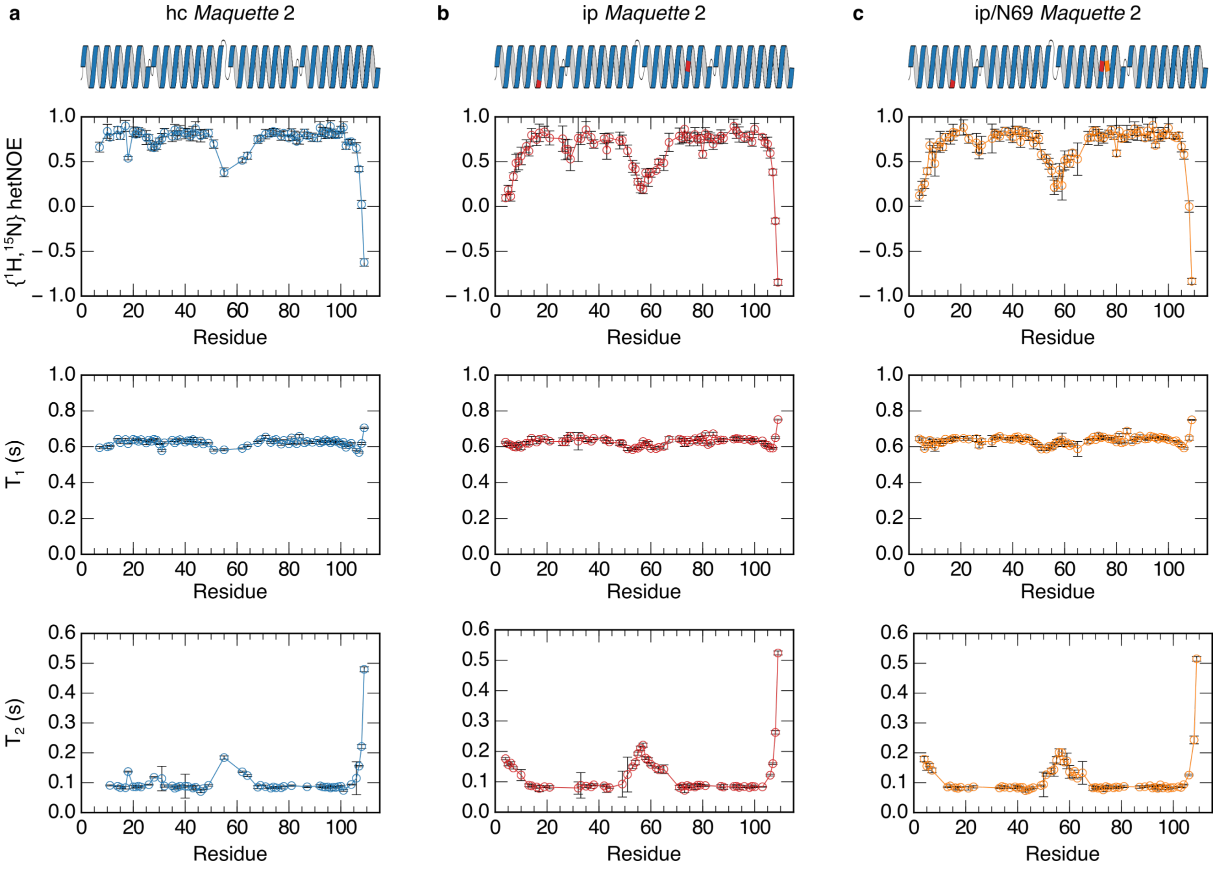

Supplementary Figure 8 | Relaxation data of *Maquette* 2 models. The figure shows hetNOE, T_1, and T_2 relaxation data for **a,** hydrophobic core, **b,** ion-pair, and **c,** charge-stabilised ion-pair *Maquette* 2. The experimental error was set to two times the standard deviation of the spectral noise. Uncertainties of hetNOE, T1, T2 were estimated by 1000 Monte Carlo runs. The mean and standard deviations are plotted.

Supplementary Table 1 | Sequences of the *de novo*-protein *Maquettes*.

Construct	Sequence
***Maquette* 1**<br>**Hydrophobic core (hc)**<br>Scaffold source Ref. 2	G EIWKQFE DALQKFE EALNQFEDLKQL GGSGSGSGG<br>EIWKQFE DALQKFE EALNQFEDLKQL GGSGSGSGG<br>EIWKQFE DALQKFE EALNQFEDLKQL GGSGSGSGG<br>EIWKQFE DALQKFE EALNQFEDLKQL
***Maquette* 1**<br>**Buried positive charge (F49K)**	G EIWKQFE DALQKFE EALNQFEDLKQL GGSGSGSGG<br>EIWKQFE DALQK**K**E EALNQFEDLKQL GGSGSGSGG<br>EIWKQFE DALQKFE EALNQFEDLKQL GGSGSGSGG<br>EIWKQFE DALQKFE EALNQFEDLKQL
***Maquette* 1**<br>**Buried negative charge (F49E)**	G EIWKQFE DALQKFE EALNQFEDLKQL GGSGSGSGG<br>EIWKQFE DALQK**E**E EALNQFEDLKQL GGSGSGSGG<br>EIWKQFE DALQKFE EALNQFEDLKQL GGSGSGSGG<br>EIWKQFE DALQKFE EALNQFEDLKQL
***Maquette* 1**<br>**Buried ion-pair (1ip/F49E/F84K)**	G EIWKQFE DALQKFE EALNQFEDLKQL GGSGSGSGG<br>EIWKQFE DALQK**E**E EALNQFEDLKQL GGSGSGSGG<br>EIWKQFE DALQK**K**E EALNQFEDLKQL GGSGSGSGG<br>EIWKQFE DALQKFE EALNQFEDLKQL
***Maquette* 1**<br>**Buried ion-pair with reversed polarity (1ip$_{rev}$/F49K/F84E)**	G EIWKQFE DALQKFE EALNQFEDLKQL GGSGSGSGG<br>EIWKQFE DALQK**K**E EALNQFEDLKQL GGSGSGSGG<br>EIWKQFE DALQK**E**E EALNQFEDLKQL GGSGSGSGG<br>EIWKQFE DALQKFE EALNQFEDLKQL
***Maquette* 2**<br>**Hydrophobic core (hc)**<br>Scaffold source Ref. 3	S EFEKLRQ TGDELVQ AFQRLREIFDK GD<br>DDSLEQV LEEIEEL IQKHRQLFDNR QEAA<br>DTEAAKQ GDQWVQL FQRFREAIDK GD<br>KDSLEQL LEELEQA LQKIRELAEKKN
***Maquette* 2**<br>**Buried ion-pair (1ip/F17E/F72K)**	S EFEKLRQ TGDELVQ A**E**QRLREIFDK GD<br>DDSLEQV LEEIEEL IQKHRQLFDNR QEAA<br>DTEAAKQ GDQWVQL **K**QRFREAIDK GD<br>KDSLEQL LEELEQA LQKIRELAEKKN
***Maquette* 2**<br>**F17E**	S EFEKLRQ TGDELVQ A**E**QRLREIFDK GD<br>DDSLEQV LEEIEEL IQKHRQLFDNR QEAA<br>DTEAAKQ GDQWVQL KQRFREAIDK GD<br>KDSLEQL LEELEQA LQKIRELAEKKN
***Maquette* 2**<br>**F72K**	S EFEKLRQ TGDELVQ AEQRLREIFDK GD<br>DDSLEQV LEEIEEL IQKHRQLFDNR QEAA<br>DTEAAKQ GDQWVQL **K**QRFREAIDK GD<br>KDSLEQL LEELEQA LQKIRELAEKKN
***Maquette* 2**<br>**Charge stabilized ion-pair (1ip/V69N)**	S EFEKLRQ TGDELVQ A**E**QRLREIFDK GD<br>DDSLEQV LEEIEEL IQKHRQLFDNR QEAA<br>DTEAAKQ GDQW**N**QL **K**QRFREAIDK GD<br>KDSLEQL LEELEQA LQKIRELAEKKN
***Maquette* 2**<br>**Charge stabilized ion-pair (1ip/I43Y)**	S EFEKLRQ TGDELVQ A**E**QRLREIFDK GD<br>DDSLEQV LEEIEEL **Y**QKHRQLFDNR QEAA<br>DTEAAKQ GDQWVQL **K**QRFREAIDK GD<br>KDSLEQL LEELEQA LQKIRELAEKKN
***Maquette* 2**<br>**Charge stabilized ion-pair (1ip/I39Q)**	S EFEKLRQ TGDELVQ A**E**QRLREIFDK GD<br>DDSLEQV LEE**Q**EEL IQKHRQLFDNR QEAA<br>DTEAAKQ GDQWVQL **K**QRFREAIDK GD<br>KDSLEQL LEELEQA LQKIRELAEKKN
***Maquette* 2**<br>**Charge stabilized ion-pair (1ip/L94N)**	S EFEKLRQ TGDELVQ A**E**QRLREIFDK GD<br>DDSLEQV LEEIEEL IQKHRQLFDNR QEAA<br>DTEAAKQ GDQWVQL **K**QRFREAIDK GD<br>KDSLEQL LEE**N**EQA LQKIRELAEKKN
***Maquette* 2**<br>**Charge stabilized ion-pair (1ip/L91H)**	S EFEKLRQ TGDELVQ A**E**QRLREIFDK GD<br>DDSLEQV LEEIEEL IQKHRQLFDNR QEAA<br>DTEAAKQ GDQWVQL **K**QRFREAIDK GD<br>KDSLEQL **H**EELEQA LQKIRELAEKKN
***Maquette* 2**<br>**Charge stabilized ion-pair (1ip/R76S)**	S EFEKLRQ TGDELVQ A**E**QRLREIFDK GD<br>DDSLEQV LEEIEEL IQKHRQLFDNR QEAA<br>DTEAAKQ GDQWVQL **K**QRF**S**EAIDK GD<br>KDSLEQL LEELEQA LQKIRELAEKKN
***Maquette* 2**<br>**hydrophilic ion-pair (F50K/I101E)**	S EFEKLRQ TGDELVQ AFQRLREIFDK GD<br>DDSLEQV LEEIEEL IQKHRQL**K**DNR QEAA<br>DTEAAKQ GDQWVQL FQRFREAIDK GD<br>KDSLEQL LEELEQA LQK**E**RELAEKKN
**5VJT**	GSPELRQ EHQQLAQ EFQQLLQEIQQL GRELLKG ELQGIKQ LREASEK ARN<br>PEKKSVL QKILEDE EKHIELLETLQQ TGQEAQQ LLQELQQ TGQELWQL GGSGG<br>PELRQKH QQLAQKI QQLLQKHQQLGA KILEDEE KHIELLE TIL GGSGG<br>DELRELL KGELQGI KQYRELQQLGQK AQQLVQK LQQTGQK LWQLG
***Maquette* 3**<br>**Stabilized charged cluster (2ip/4Q)**	M ASPELRQ EFQQLIQ EFQQLLQEIQQL IRELLKI **K**LQIIKQ LREASEK ARN<br>PEKKSVL QK**Q**LEL**E** EK**Q**IELLETLQQ TAQEAQQ LLQELQQ TGQELWQL GGSGG<br>PELRQKF QQLAQKI QQLLQKFQQLVA K**Q**LED**E**E KFIELLE TIL GGSGG<br>DELRELL KG**K**LQVI KQ**Q**RELLQLVQK AQQLVQK LQQTGQK LW

***Maquette* 3**<br>**Stabilized charged cluster**<br>**(2ip/Q61A/Q68A)**	M	ASPELRQ EFQQLIQ EFQQLLQEIQQL IRELLKI **K**LQIIKQ LREASEK<br>PEKKSVL QK**A**LEL**E** EK**A**IELLETLQQ TAQEAQQ LLQELQQ TGQELWQL<br>PELRQKF QQLAQKI QQLLQKFQQLVA K**Q**LED**E**E KFIELLE TIL<br>DELRELL KG**K**LQVI KQ**Q**RELLQLVQK AQQLVQK LQQTGQK LW	ARN<br>GGSGG<br>GGSGG

Supplementary Table 2 | NMR and refinement statistics of *Maquette* 2.

	Maquette 2/ip		*Maquette* 2/ip closed	
	**CYANA Result**	**Energy Minimized[b]**	**CYANA Result**	**Energy Minimized[b]**
**Automated NOE assignment[a]**				
^{15}N-resolved NOESY cross-peaks	1758		1758	
^{13}C-resolved aliphatic NOESY cross-peaks	1938		1938	
^{13}C-resolved aromatic NOESY cross-peaks	237		237	
Total no. of NOESY cross-peaks	3933 (100%)		3933 (100%)	
Assigned cross-peaks	2861 (72.7%)		2854 (72.6%)	
Unassigned cross-peaks	1072 (27.3%)		1079 (27.4%)	
**Structural restraints**				
Assigned NOE distance restraints	1918 (100%)		1923 (100%)	
Short range ($\|i-j\| \leq 1$)	1109 (57.8%)		1119 (58.2%)	
Medium-range ($1 < \|i-j\| < 5$)	533 (27.8%)		512 (26.6%)	
Long-range ($\|i-j\| \geq 5$)	276 (14.4%)		292 (15.2%)	
Dihedral angle restraints TALOS-N (ϕ/ψ)	190		190	
**Structure statistics**				
Average CYANA target function value (Å^2)	2.25 ± 0.41	1.80 ± 0.27	9.48 ± 16.27	2.23 ± 0.45
Average AMBER energy (kcal/mol)	-3829.55 ± 81.08	-4847.10 ± 132.53	-3863.46 ± 159.03	-4850.34 ± 148.53
**Restraint violations**				
Max. distance restraint violation (Å)	0.95	0.12	2.76	0.13
No. of violated distance restraints >0.2 Å	13	0	10	1
Max. dihedral angle restraint violation (°)	6.67	3.59	5.68	3.41
No. of violated dihedral angle restraints >5°	4	1	4	1
**Ramachandran plot**				
Residues in most favored regions	92.1%	93.5%	92.6%	93.4%
Residues in additionally allowed regions	7.8%	6.2%	7.3%	6.3%
Residues in generously allowed regions	0.1%	0.3%	0.1%	0.3%
Residues in disallowed regions	0%	0%	0%	0%
**RMSD (residues 1-109)****				
Average backbone RMSD to mean (Å)	0.75 ± 0.16	0.76 ± 0.15	0.75 ± 0.19	0.77 ± 0.18
Average heavy atom RMSD to mean (Å)	1.27 ± 0.14	1.29 ± 0.13	1.23 ± 0.18	1.27 ± 0.17

[a]Using structure calculation functionalities of CYANA.
[b]After restrained energy minimization with OPALp.
**Pairwise r.m.s. deviation was calculated among 20 structures.

Supplementary Table 3 | Crystallographic data collection and refinement statistics of *Maquette* 3.

	***Maquette* 3**
**Data collection**	
Space group	P1
Cell dimensions	
a, b, c (Å)	62.7, 65.9, 68.3
α, β, γ (°)	89.9, 90.3, 117.5
Resolution (Å)	30-1.85 (1.85-1.75)
R_{merge}	0.051 (0.545)
$I / \sigma I$	11.3 (2.1)
Completeness (%)	97.6 (98.5)
Redundancy	3.4 (3.6)
**Refinement**	
Resolution (Å)	30-1.85
No. reflections	76730
R_{work} / R_{free}	0.189 / 0.223
No. atoms	
Protein	6488
Ligand/ion	2
Water	123
B-factors	
Protein	45
Ligand/ion	41
Water	46
R.m.s. deviations	
Bond lengths (Å)	0.003
Bond angles (°)	1.1

*Data have been collected from two crystals, values in parentheses are for highest-resolution shell.

Supplementary Table 4 | Summary of performed MD simulations.

Construct	Length (ns)	#Replicas	System size (atoms)
***Maquette* 1**<br>**Hydrophobic core (hc)**	200	2	43,912
***Maquette* 1**<br>**Buried positive charge (Lys49)**	200	2	43,919
***Maquette* 1**<br>**Buried negative charge (Glu49)**	200	2	43,908
***Maquette* 1**<br>**Buried ion-pair Lys49/Glu49 (1ip)**	200	2	43,915
***Maquette* 1**<br>**Buried ion-pair with reversed polarity ($1ip_{rev}$)**	200	2	43,915
***Maquette* 2**<br>**Hydrophobic core (hc)**	200	2	66,932
***Maquette* 2**<br>**Buried ion-pair (1ip)**	200	3	44,429-65,030
***Maquette* 2**<br>**hydrophilic ion-pair (Lys50/Glu101)**	200	2	51,953
***Maquette* 2**<br>**Lys72/Glu17 NMR structure**	200	2	48,057
***Maquette* 2**<br>**Lys72/Glu72/N69**	200	2	48,059
***Maquette* 3**<br>**Stabilized charged cluster (2ip/4Q)**	200	2	289,717
**Total simulation time (ns)**	4600		

Supplementary Table 5 | Summary of primers used.

Primer name	Primer sequence
K72F_1	TGATCAATGGGTGCAGCTGTTCCAACGTTTTCGTGAAGCGATC
K72F_2	GATCGCTTCACGAAAACGTTGGAACAGCTGCACCCATTGATCA
E17F_1	GAGCTGGTGCAGGCGTTCCAACGTCTGCGTGAGATCTTC
E17F_2<br>L91H_1	GAAGATCTCACGCAGACGTTGGAACGCCTGCACCAGCTC<br>CAGCCTGGAACAGCTGCACGAGGAACTGGAGCAGGCG
L91H_2	CGCCTGCTCCAGTTCCTCGTGCAGCTGTTCCAGGCTG
R76S_1	GCAGCTGAAACAACGTTTTAGTGAAGCGATCGACAAGGGCG
R76S_2	CGCCCTTGTCGATCGCTTCACTAAAACGTTGTTTCAGCTGC
I43Y_1	GGAGGAAATCGAGGAACTGTATCAGAAACACCGTCAACTGTTC
I43Y_2	GAACAGTTGACGGTGTTTCTGATACAGTTCCTCGATTTCCTCC
I39Q_1	GAGCAGGTTCTGGAGGAACAGGAGGAACTGATTCAGAAACACC
I39Q_2	GGTGTTTCTGAATCAGTTCCTCCTGTTCCTCCAGAACCTGCTC

Supplementary Table 6 | DNA sequences of the *de novo*-protein *Maquettes*.

Construct	DNA Sequence
***Maquette* 1**<br>**Hydrophobic core (hc)**<br>Scaffold source Ref. 2	ggtgagatttggaagcaatttgaagatgcgctgcagaagtttgaagaggcg<br>ctgaaccagtttgaagacctgaagcaactgggtggcagcggtagcggcagc<br>ggtggcgagatctggaagcagtttgaagacgcgctgcaaaagtttgaggaa<br>gcgctgaaccagttcgaggatctgaaacaactgggtggcagcggtagcggc<br>agcggtggcgagatatggaagcagttcgaagacgcgctgcaaaaatttgag<br>gaagcgctgaatcaattcgaagatctgaaacagctgggtggcagcggtagc<br>ggcagcggtggcgagatttggaaacagtttgaagacgcgctgcaaaagttt<br>gaagaggcgctgaaccaatttgaagacctgaaacaactg
***Maquette* 1**<br>**Buried positive charge (F49K)**	ggtgagatttggaagcaatttgaagatgcgctgcagaagtttgaagaggcg<br>ctgaaccagtttgaagacctgaagcaactgggtggcagcggtagcggcagc<br>ggtggcgagatctggaagcagtttgaagacgcgctgcaaaagaaagaggaa<br>gcgctgaaccagttcgaggatctgaaacaactgggtggcagcggtagcggc<br>agcggtggcgagatatggaagcagttcgaagacgcgctgcaaaaatttgag<br>gaagcgctgaatcaattcgaagatctgaaacagctgggtggcagcggtagc<br>ggcagcggtggcgagatttggaaacagtttgaagacgcgctgcaaaagttt<br>gaagaggcgctgaaccaatttgaagacctgaaacaactg
***Maquette* 1**<br>**Buried negative charge (F49E)**	ggtgagatttggaagcagtttgaagatgcgctgcagaagtttgaagaggcg<br>ctgaaccaatttgaagacctgaagcaactgggtggcagcggtagcggcagc<br>ggtggcgaaatctggaagcagtttgaggacgcgctgcaaaaagaggaagag<br>gcgctgaaccagttcgaagatctgaaacagctgggtggcagcggtagcggc<br>agcggtggcgaaatatggaagcagttcgaagatgcgctgcaaaaattcgaa<br>gaggcgctgaatcagttcgaggacttaaaacagctgggtggcagcggtagc<br>ggcagcggtggcgaaatttggaaacagtttgaggacgcgctgcaaaagttt<br>gaagaggcgctgaaccaatttgaagacctgaaacaactg
***Maquette* 1**<br>**Buried ion-pair (1ip/F49E/F84K)**	ggtgagatttggaagcaatttgaagatgcgctgcagaagtttgaagaggcg<br>ctgaaccagtttgaagacctgaagcaactgggtggcagcggtagcggcagc<br>ggtggcgaaatctggaagcagttcgaggacgcgctgcaaaaagaggaagag<br>gcgctgaatcaatttgaagatctgaaacagctgggtggcagcggtagcggc<br>agcggtggcgaaatatggaaacaatttgaggacgcgctgcaaaagaaagaa<br>gaggcgctgaatcagttcgaggacttaaaacagctgggtggcagcggtagc<br>ggcagcggtggcgaaatttggaaacagttcgaggacgcgctgcaaaagttt<br>gaagaggcgctgaaccaatttgaggacctgaaacaactg
***Maquette* 1**<br>**Buried ion-pair with reversed polarity ($1ip_{rev}$/F49K/F84E)**	ggtgagatttggaagcaatttgaagatgcgctgcagaagtttgaagaggcg<br>ctgaaccagtttgaagacctgaagcaactgggtggcagcggtagcggcagc<br>ggtggcgaaatctggaagcagtttgaggacgcgctgcaaaagaaagaggaa<br>gcgctgaaccagttcgaagatctgaaacaactgggtggcagcggtagcggc<br>agcggtggcgaaatatggaaacaattcgaagacgcgctgcaaaaagaggaa<br>gaggcgctgaatcaattcgaggatctgaaacagctgggtggcagcggtagc<br>ggcagcggtggcgaaatttggaagcagttcgaggacgcgctgcaaaaattc<br>gaagaggcgctgaatcagttcgaggacctgaaacaactg
***Maquette* 2**<br>**Hydrophobic core (hc)**<br>Scaffold source Ref. 3	agcgagtttgaaaagctgcgtcaaaccggtgatgagctggtgcaggcgttc<br>caacgtctgcgtgaaatctttgacaaaggcgacgatgacagcctggaacag<br>gttctggaggaaatcgaggaactgattcagaagcaccgtcaactgttcgat<br>aaccgtcaagaggcggcggacaccgaagcggcgaaacagggtgatcaatgg<br>gtgcagctgttccaacgttttcgtgaggcgatcgacaagggcgataaagac<br>agcctggagcagctgctggaggaactggaacaggcgctgcaaaagattcgt<br>gagctggcggaaaagaaaaac
***Maquette* 2**<br>**Buried ion-pair (1ip/F17E/F72K)**	agcgagtttgaaaagctgcgtcaaaccggtgatgagctggtgcaggcggaa<br>caacgtctgcgtgagatcttcgacaagggcgacgatgacagcctggagcag<br>gttctggaggaaatcgaggaactgattcagaaacaccgtcaactgttcgat<br>aaccgtcaagaagcggcggacaccgaggcggcgaagcagggtgatcaatgg<br>gtgcagctgaaacaacgttttcgtgaagcgatcgacaagggcgataaagac<br>agcctggaacagctgctggaggaactggagcaggcgctgcaaaaaattcgt<br>gagctggcggaaaagaaaaac
***Maquette* 2**<br>**F17E**	agcgagtttgaaaagctgcgtcaaaccggtgatgagctggtgcaggcggaa<br>caacgtctgcgtgagatcttcgacaagggcgacgatgacagcctggagcag<br>gttctggaggaaatcgaggaactgattcagaaacaccgtcaactgttcgat<br>aaccgtcaagaagcggcggacaccgaggcggcgaagcagggtgatcaatgg<br>gtgcagctgttccaacgttttcgtgaagcgatcgacaagggcgataaagac<br>agcctggaacagctgctggaggaactggagcaggcgctgcaaaaaattcgt<br>gagctggcggaaaagaaaaac
***Maquette* 2**<br>**F72K**	agcgagtttgaaaagctgcgtcaaaccggtgatgagctggtgcaggcgttc<br>caacgtctgcgtgagatcttcgacaagggcgacgatgacagcctggagcag<br>gttctggaggaaatcgaggaactgattcagaaacaccgtcaactgttcgat<br>aaccgtcaagaagcggcggacaccgaggcggcgaagcagggtgatcaatgg<br>gtgcagctgaaacaacgttttcgtgaagcgatcgacaagggcgataaagac<br>agcctggaacagctgctggaggaactggagcaggcgctgcaaaaaattcgt<br>gagctggcggaaaagaaaaac
***Maquette* 2**<br>**Charge stabilized ion-pair (1ip/V69N)**	agcgagtttgaaaagctgcgtcaaaccggtgatgagctggtgcaggcggaa<br>caacgtctgcgtgagatcttcgacaagggcgacgatgacagcctggagcag<br>gttctggaggaaatcgaggaactgattcagaaacaccgtcaactgttcgat<br>aaccgtcaagaagcggcggacaccgaggcggcgaagcagggtgatcaatgg<br>aaccagctgaaacaacgttttcgtgaagcgatcgacaagggcgataaagac<br>agcctggaacagctgctggaggaactggagcaggcgctgcaaaaaattcgt<br>gagctggcggaaaagaaaaac

***Maquette* 2**<br>**Charge stabilized ion-pair**<br>**(1ip/I43Y)**	agcgagtttgaaaagctgcgtcaaaccggtgatgagctggtgcaggcggaa<br>caacgtctgcgtgagatcttcgacaagggcgacgatgacagcctggagcag<br>gttctggaggaaatcgaggaactgtatcagaaacaccgtcaactgttcgat<br>aaccgtcaagaagcggcggacaccgaggcggcgaagcagggtgatcaatgg<br>gtgcagctgaaacaacgttttcgtgaagcgatcgacaagggcgataaagac<br>agcctggaacagctgctggaggaactggagcaggcgctgcaaaaaattcgt<br>gagctggcggaaaagaaaaac
***Maquette* 2**<br>**Charge stabilized ion-pair**<br>**(1ip/I39Q)**	agcgagtttgaaaagctgcgtcaaaccggtgatgagctggtgcaggcggaa<br>caacgtctgcgtgagatcttcgacaagggcgacgatgacagcctggagcag<br>gttctggaggaacaggaggaactgattcagaaacaccgtcaactgttcgat<br>aaccgtcaagaagcggcggacaccgaggcggcgaagcagggtgatcaatgg<br>gtgcagctgaaacaacgttttcgtgaagcgatcgacaagggcgataaagac<br>agcctggaacagctgctggaggaactggagcaggcgctgcaaaaaattcgt<br>gagctggcggaaaagaaaaac
***Maquette* 2**<br>**Charge stabilized ion-pair**<br>**(1ip/L94N)**	agcgagtttgaaaagctgcgtcaaaccggtgatgagctggtgcaggcggaa<br>caacgtctgcgtgagatcttcgacaagggcgacgatgacagcctggagcag<br>gttctggaggaaatcgaggaactgattcagaaacaccgtcaactgttcgat<br>aaccgtcaagaagcggcggacaccgaggcggcgaagcagggtgatcaatgg<br>gtgcagctgaaacaacgttttcgtgaagcgatcgacaagggcgataaagac<br>agcctggaacagctgctggaggaaaacgagcaggcgctgcaaaaaattcgt<br>gagctggcggaaaagaaaaac
***Maquette* 2**<br>**Charge stabilized ion-pair**<br>**(1ip/L91H)**	agcgagtttgaaaagctgcgtcaaaccggtgatgagctggtgcaggcggaa<br>caacgtctgcgtgagatcttcgacaagggcgacgatgacagcctggagcag<br>gttctggaggaaatcgaggaactgattcagaaacaccgtcaactgttcgat<br>aaccgtcaagaagcggcggacaccgaggcggcgaagcagggtgatcaatgg<br>gtgcagctgaaacaacgttttcgtgaagcgatcgacaagggcgataaagac<br>agcctggaacagctgcacgaggaactggagcaggcgctgcaaaaaattcgt<br>gagctggcggaaaagaaaaac
***Maquette* 2**<br>**Charge stabilized ion-pair**<br>**(1ip/R76S)**	agcgagtttgaaaagctgcgtcaaaccggtgatgagctggtgcaggcggaa<br>caacgtctgcgtgagatcttcgacaagggcgacgatgacagcctggagcag<br>gttctggaggaaatcgaggaactgattcagaaacaccgtcaactgttcgat<br>aaccgtcaagaagcggcggacaccgaggcggcgaagcagggtgatcaatgg<br>gtgcagctgaaacaacgttttagtgaagcgatcgacaagggcgataaagac<br>agcctggaacagctgctggaggaactggagcaggcgctgcaaaaaattcgt<br>gagctggcggaaaagaaaaac
***Maquette* 2**<br>**hydrophilic ion-pair**<br>**(F50K/I101E)**	agcgagtttgaaaagctgcgtcaaaccggtgatgagctggtgcaggcgttc<br>caacgtctgcgtgaaatctttgacaaaggcgacgatgacagcctggaacag<br>gttctggaggaaatcgaggaactgattcagaagcaccgtcaactgaaagat<br>aaccgtcaagaggcggcggacaccgaagcggcgaagcagggtgatcaatgg<br>gtgcagctgttccaacgttttcgtgaggcgattgacaagggcgataaagac<br>agcctggagcagctgctggaggaactggaacaggcgctgcaaaaagagcgt<br>gaactggcggaaaagaaaaac
***Maquette* 3**<br>**Stabilized charged cluster**<br>**(2ip/4Q)**	atggcgagcccggagctgcgtcaggaattccagcaactgatccaagagttt<br>cagcaactgctgcaggaaatccagcaactgattcgtgagctgctgaagatt<br>aaactgcagatcattaagcaactgcgtgaggcgagcgaaaaagcgcgtaac<br>ccggagaagaaaagcgtgctgcagaagcaactggaactggaggaaaaacag<br>atcgagctgctggaaaccctgcagcaaaccgcgcaagaagcgcagcaactg<br>ctgcaggagctgcagcaaaccggtcaggaactgtggcagctgggtggcagc<br>ggtggcccggagctgcgtcaaaagttccagcaactggcgcagaaaatccag<br>caactgctgcaaaagtttcagcaactggtggcgaaacagctggaagacgag<br>gaaaagttcattgaactgctggaaaccattctgggtggcagcggtggcgat<br>gagctgcgtgaactgctgaagggcaaactgcaggttattaagcagcaacgt<br>gagctgctgcagctggtgcaaaaagcgcagcaactggttcagaagctgcaa<br>cagaccggtcagaaactgtgg
***Maquette* 3**<br>**Stabilized charged cluster**<br>**(2ip/Q61A/Q68A)**	atggcgagcccggagctgcgtcaggaattccagcaactgatccaagagttt<br>cagcaactgctgcaggaaatccagcaactgattcgtgagctgctgaagatt<br>aaactgcagatcattaagcaactgcgtgaggcgagcgaaaaagcgcgtaac<br>ccggagaagaaaagcgtgctgcagaaggcgctggaactggaggaaaaagcg<br>atcgagctgctggaaaccctgcagcaaaccgcgcaagaagcgcagcaactg<br>ctgcaggagctgcagcaaaccggtcaggaactgtggcagctgggtggcagc<br>ggtggcccggagctgcgtcaaaagttccagcaactggcgcagaaaatccag<br>caactgctgcaaaagtttcagcaactggtggcgaaacagctggaagacgag<br>gaaaagttcattgaactgctggaaaccattctgggtggcagcggtggcgat<br>gagctgcgtgaactgctgaagggcaaactgcaggttattaagcagcaacgt<br>gagctgctgcagctggtgcaaaaagcgcagcaactggttcagaagctgcaa<br>cagaccggtcagaaactgtgg

Supplementary Table 7 | Buffers used for protein purifications of *de novo*-protein *Maquettes*.

Construct	Buffer
Maquette 1/*Maquette* 3	20 mM Tris pH 8, 100 mM NaCl
	20 mM Tris pH 8, 50 mM NaCl, 1 mM EDTA (+ 2 mM DTT for TEV)
Maquette 2	50 mM NaPi pH 7, 150 mM NaCl
	50 mM Tris pH 8, 1 mM EDTA (+ 2 mM DTT for TEV)
	50 mM NaPi pH 7.5, 100 mM NaCl

Supplementary References

1. Lukasz P. Kozlowski, Proteome-pI: proteome isoelectric point database, *Nucleic Acids Research*. **45**, D1112–D1116 (2017).
2. Farid, T. A. *et al.* Elementary tetrahelical protein design for diverse oxidoreductase functions. *Nat. Chem. Biol.* **9**, 826–833 (2013).
3. Polizzi, N. F. *et al.* De novo design of a hyperstable non-natural protein-ligand complex with sub-Å accuracy. *Nat. Chem.* **9**, 1157–1164 (2017).
